# Antimicrobial Metabolites Produced by *Penicillium mallochii* CCH01 Isolated From the Gut of *Ectropis oblique*, Cultivated in the Presence of a Histone Deacetylase Inhibitor

**DOI:** 10.3389/fmicb.2019.02186

**Published:** 2019-10-02

**Authors:** Shuxiang Zhang, Han Fang, Caiping Yin, Chaoling Wei, Jingwei Hu, Yinglao Zhang

**Affiliations:** ^1^School of Life Sciences, Anhui Agricultural University, Hefei, China; ^2^State Key Laboratory of Tea Plant Biology and Utilization, Anhui Agricultural University, Hefei, China; ^3^Biotechnology Center, Anhui Agricultural University, Hefei, China

**Keywords:** gut fungus, *Ectropis oblique*, *Penicillium mallochii* CCH01, suberoylanilide hydroxamic acid, antimicrobial activities

## Abstract

Three chemical epigenetic modifiers [5-azacytidine, nicotinamide, and suberoylanilide hydroxamic acid (SAHA)] were applied to induce the metabolites of *Penicillium mallochii* CCH01, a fungus isolated from the gut of *Ectropis oblique*. Metabolite profiles of *P. mallochii* CCH01 were obviously changed by SAHA treatment. Four metabolites (**1**–**4**), including two new natural sclerotioramine derivatives, isochromophilone XIV (**1**) and isochromophilone XV (**2**), and two known compounds, sclerotioramine (**3**) and (+)-sclerotiorin (**4**), were isolated and purified from *P. mallochii* CCH01 treated with SAHA. Their structures were determined by spectroscopic analyzes. Anti-phytopathogenic activities of the isolated compounds **1**–**4** were investigated under laboratory conditions, and compound **4** showed broad and important inhibition activities against *Curvularia lunata* (IC_50_ = 2.1 μg/mL), *Curvularia clavata* (IC_50_ = 21.0 μg/mL), *Fusarium oxysporum* f. sp. *Mornordica* (IC_50_ = 40.4 μg/mL), and *Botryosphaeria dothidea* (IC_50_ = 27.8 μg/mL), which were comparable to those of referenced cycloheximide, with IC_50_ values of 0.3, 5.0, 12.4, and 15.3 μg/mL, respectively. Ingredients **2** and **3** showed selective and potent activities against *Colletotrichum graminicola* with IC_50_ values of 29.9 and 9.7 μg/mL, respectively. Furthermore, the antibacterial bioassays showed that compounds **3** and **4** exhibited strong inhibition activities against *Bacillus subtilis*, with disc diameters of zone of inhibition (ZOI) of 9.1 mm for both compounds, which were a bit weaker than that of referenced gentamycin with a ZOI of 10.8 mm. Additionally, the new metabolite **1** showed a promising activity against *Candida albicans* (ZOI = 10.5 mm), comparable to that of positive amphotericin B with a ZOI of 23.2 mm. The present results suggest that chemical epigenetic modifier induction was a promising approach to obtaining antimicrobial metabolites encoded by silent biosynthetic genes of *P. mallochii*.

## Introduction

Fungi produce a wealth of valuable natural products like alkaloids, phenolic acids, flavonoids, quinones, terpenoids, steroids, benzopyranones, tetralones, xanthones, and peptides with a remarkable range of biological activities in medicine and agriculture. However, sequenced fungi genomes reveal that there are far more secondary metabolite biosynthesis gene clusters than were clear in chemical studies (Cichewicz, 2010; Sanchez et al., 2012; Ochi and Hosaka, 2013). This suggests that the potential of generating untapped chemical of fungi metabolite under standard laboratory conditions is limited, as a result of transcriptional suppression of numerous biosynthetic gene clusters (BGCs). To improve the rate of discovering novel bioactive compounds, epigenetic perturbation approaches are used to activate or modify these silent BGCs. There have been many successful examples of the use of DNA methyltransferase (DMT) inhibitors to recall cryptic biosynthetic clusters in fungi (Asai et al., 2012; He et al., 2015; Chen et al., 2016; Yu et al., 2019). Like DMT inhibitors, histone deacetylase (HDAC) inhibitors were often employed to manipulate the fungal epigenome, too. Exposing cultures of *Graphiopsis chlorocephala* to the NAD^+^-dependent HDAC inhibitor, nicotinamide triggered dramatic changes in the fungal metabolite profile, enabling the isolation of new polyketides (Asai et al., 2012). Cultivating *Eupenicillium* sp. LG41 in the presence of the same HDAC inhibitor led to enhanced production of two new decalin-containing compounds, eupenicinicols C and D (Li et al., 2017). Similarly, treating *Daldinia* sp. (Du et al., 2014), *Aspergillus wentii* (Miao et al., 2014), *Chalara* sp. 6661 (Adpressa et al., 2017), and *Cladosporium sphaerospermum* with the Zn (II) NAD^+^-independent HDAC inhibitor [suberoylanilide hydroxamic acid (SAHA)] contributed to the discovery of new polyketides, diterpenes, xanthones, and tetramic acids (Zhang et al., 2018).

We have been devoting our efforts toward discovering bioactive compounds produced by insect-associated fungi (Zhang et al., 2008, 2011; Li et al., 2014). In order to maximize the opportunity for detecting novel secondary metabolites, three chemical epigenetic modifiers were employed to stimulate the metabolite production of *Penicillium mallochii* CCH01 from the gut of *Ectropis oblique*, a pest living on tea plants. We found that metabolite profiles of the fungal strain showed obvious changes, when treated with SAHA. In this paper, we report the details of the isolation, structure elucidation, and biological activities of the metabolites produced by *P. mallochii* CCH01 cultivated in the presence of SAHA.

## Materials and Methods

### Isolation and Identification of Fungus From *E. oblique*

The *E. oblique* was collected from the tea plantation of Anhui Agricultural University, Hefei, China (latitude: 31.86 N, longitude: 117.27 E, altitude: 20 m above mean sea level). Insects (eight individuals) were kept without food for 1 day and then dissected under sterile conditions. Individual guts were suspended in a vial of 1 ml of 1 × phosphate buffer and further diluted at proportions of 10^–1^, 10^–2^, 10^–3^ in 1 × phosphate buffer. One hundred microliters of the diluted gut solutions was plated on PDA (20 g L^–1^ potato, 20 g L^–1^ glucose, and 20 g L^–1^ agar) plates supplemented with potassium dichromate (50 mg L^–1^) and nalidixic acid (25 mg L^–1^). The plates were incubated at 28°C for 4 days for fungus growth and any fungal colonies that formed were sub-cultured on new PDA medium to obtain pure cultures. The isolated strain CCH01 was deposited at the China Center for Type Culture Collection (CCTCC) as CCTCC M2018235, and also preserved on PDA slants at 4°C until use. Morphological character and molecular identification (based on the DNA sequence of ITS region using ITS1 and ITS4 primers after PCR amplification) were implemented to identify the strain.

### Effect of Epigenetic Modifying Compounds on the Metabolite Production of *P. mallochii* CCH01

From a 7-day-old MEA (consisting of 20 g of malt extract, 20 g of sucrose, 1 g of peptone, and 20 g of agar in 1 L of distilled water) culture plate, a conidial spore suspension was prepared by adding 15 mL of distilled water containing 0.2% Tween 80 (w/v) under agitation by glass rod. To study the effect of epigenetic modifiers, aliquots (2 mL) of spore suspension were added into 100 mL of ME production media (consisting of 20 g of malt extract, 20 g of sucrose, and 1 g of peptone in 1 L of distilled water) separately supplemented with DMSO-dissolved SAHA, 5-azacytidine, nicotinamide, resulting in the same final concentrations of 1 mM. Equal amounts of DMSO were added to the control group. The cultures were fermented at 28°C in a shaker rotating at 180 rpm for 10 days. The filter of each fermentation broth was extracted three times with equal amounts of EtOAc. The EtOAc extracts were fast analyzed by TLC.

### Fermentation, Extraction, and Isolation

The fungus CCH01 was grown on a ME media (twenty 1000-mL Erlenmeyer flasks, each containing 400 mL of ME media) supplemented with SAHA (in a final concentration of 1 mM) at 28°C in a shaker rotating at 180 rpm for 10 days. Total filter of fermentation broth was extracted with EtOAc (3 × 8 L) at room temperature. The EtOAc phase was evaporated *in vacuo* to afford a crude extract (14.36 g) and then chromatographed on a silica gel column eluting with a step gradient of CH_2_Cl_2_/MeOH (100:0–100:3, v/v) to give seven fractions (Fr1–Fr3). Fr1 (CH_2_Cl_2_/MeOH, 100:0, v/v) was further fractionated on a silica gel column, eluting with petroleum ether (PE)–ethyl acetate (EA) (50:1, v/v) to yield compound **4** (35 mg). Fr2 (CH_2_Cl_2_/MeOH, 100:1, v/v) was repeatedly subjected to a silica gel column (CH_2_Cl_2_/MeOH, 100:0, 100:1, v/v) to give three subfractions (R1–R3); compound **3** (48 mg) crystallized from the EtOAc solution of subfraction R1. Subfractions R2 and R3 were loaded onto a Sephadex LH-20 column (MeOH) to give compounds give compound **1** (15 mg) and **2** (20 mg), separately.

Structure elucidations of the secondary metabolites were made according to the spectroscopic analysis. The NMR spectra were recorded at 25°C with Agilent 600 MHz DD2 spectrometer NMR.

### Analysis of Compounds in Extracts by UHPLC-Q-TOF-MS

To determine that the isolated compounds were generated by SAHA, the EtOAc extracts were accurately analyzed by ultra-high-performance liquid chromatography coupled with Q-TOF mass spectrometer (Agilent Technologies, Santa Clara, CA, United States). Chromatographic analysis was performed by using a C18 reverse-phase analytical column: Phenomenex Kinetex 2.6 μ XB-C18 100A (Torrance, CA, United States). UHPLC-Q-TOF parameters were as follows: the column oven temperature was set at 40°C and the injection volume was 5 μL, with a flow rate of 0.4 mL/min. The mobile phase consisted of 0.4% acetic acid in water and 100% acetonitrile; the gradient of the latter increased linearly from 10 to 60% (v/v) at 10 min, to 100% at 15 min, to 60% at 18 min, and to 10% at 20 min. Samples were analyzed in the fast polarity switching mode at a fragmentation voltage of 175 V, over the range of *m*/*z* 100–1700. A drying gas flow rate of 11 L/min under a temperature of 350°C and a capillary voltage of 3.5 kV were used.

### Antimicrobial Activity *in vitro*

Purified metabolites were screened for their antimicrobial activity. We used nine different phytopathogenic fungi, i.e., *Colletotrichum graminicola*, *Curvularia lunata*, *Gibberella zeae*, *Curvularia clavata*, *Alternaria solani*, *Corynespora cassiicola*, *Fusarium oxysporum* f. sp. *cucumerinum*, *F. oxysporum* f. sp. *mornordicae*, and *Botryosphaeria dothidea*, and four human pathogens (*Escherichia coli*, *Staphylococcus aureus*, *Bacillus subtilis*, and *Candida albicans*) as the tested strains.

The antifungal activity *in vitro* against phytopathogenic fungi was assayed by the growth rate method (Bibi et al., 2012; Li et al., 2014), with slight modifications. Purified metabolites were dissolved in aqueous solution described previously (Yin et al., 2018) and then mixed with PDA in a Petri dish (9 cm in diameter). Cycloheximide was used as the positive control. The tested phytopathogenic fungi were inoculated onto the center of the medium and then incubated in the dark at 28°C. When the fungal mycelium reached the edges of the control dishes, the antifungal activities were calculated. The percentage of growth inhibition was calculated using the following formula:

Inhibition(%)=(1-Da/Db)×100

where Da meant the diameter of growth zone in the experimental dish (mm) and Db meant the diameter in the control dish (mm). IC_50_ values were calculated by probit analysis based on percentage of radical growth.

The disc diffusion method was applied to evaluate the antibacterial activities and anti-yeast activities of isolated metabolites. Filter paper disks with metabolite dissolved in DMSO in a concentration (30 μg/filter paper) were added to the culture medium, and the plates were incubated at 37°C (*E. coli*, *S. aureus*, and *B. subtilis*) or 28°C (*C. albicans*) for 24 h. Filter papers with DMSO, streptomycin sulfate, gentamycin sulfate, and amphotericin B were set as negative and positive controls, respectively. The zone of inhibition (ZOI) was determined by measuring the distance from the center of the disk to the end of the clear zone.

All experiments were performed in triplicate, and data were shown as mean values and standard deviation.

## Results

### Identification of the Fungus CCH01

**FIGURE 1:**
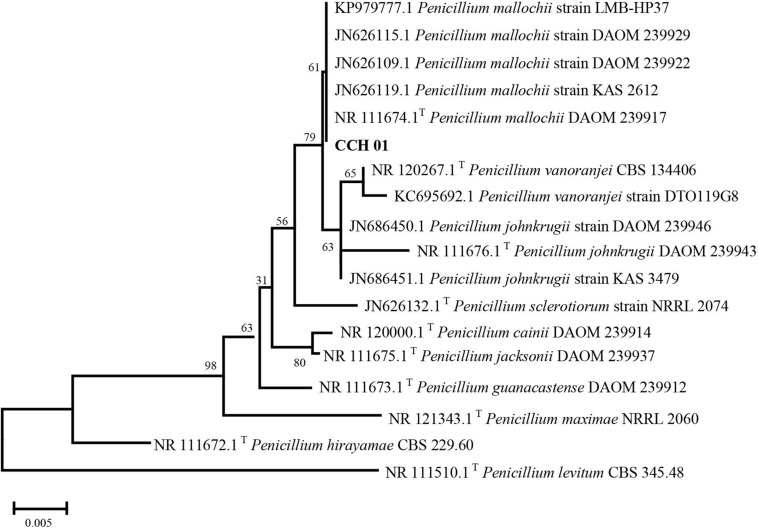
Neighbor-joining tree based on ITS nucleotide sequences.

Colonies of CCH01 on the PDA plate grew quickly at 28°C, covering the whole plate (9 cm in diameter) in 7 days. Strain CCH01 showed morphological characteristics similar to members of section *Sclerotiora* of the *Penicillium* genus, i.e., monoverticillate conidiophores, greenish gray conidial masses and orange pigment in PDA plates (Visagie et al., 2013; Supplementary Figure S1). Phylogenetic tree (Figure 1) constructed based on ITS rDNA sequences of the fungus using MEGA5 according to the neighbor-joining method indicated that CCH01 was closely related to *P. mallochii* (NR_11674.1T), with the ITS sequence similarity of 99.8%. Combined with morphological characteristics, the fungus was identified as *P. mallochii* CCH01.

### Effect of SAHA on the Metabolic Profile of *P. mallochii* CCH01

Crude EtOAc extracts obtained from both the epigenetic treated and the control (DMSO) fermentation broths of the *P. mallochii* CCH01 were compared by TLC in mobile phase (CH_2_Cl_2_/MeOH, 40:1, v/v). A UV visible spot at an Rf value of 0.3 was observed in SAHA-treated extract, which was not visible in the extracts of the culture grown without SAHA (Supplementary Figure S2).

### Isolation of Secondary Metabolites From *P. mallochii* CCH01

**FIGURE 2:**
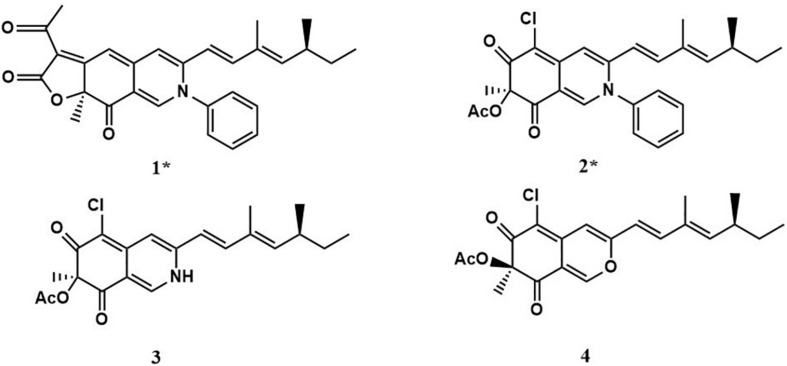
Compounds (**1**–**4**) isolated from *P. mallochii* CCH01 treated with SAHA.

Two new compounds (**1** and **2**), along with two known metabolites (**3** and **4**) were isolated from the culture of *P. mallochii* CCH01 treated with SAHA. Chemical structures of compounds **1**–**4** are shown in Figure 2.

Compound **1** (isochromophilone XIV) was obtained as a purple powder, and its molecular formula C_29_H_29_NO_4_ was deduced from HRESIMES ion peak at *m*/*z* 456.2196 [M + H]^+^, which was consistent with ^1^H and ^13^C NMR data (Table 1 and Supplementary Figures S3–S9). ^13^C NMR data were similar to those of isochromophilone IV (Kanokmedhakul et al., 2006), except ethyl signal (δ*_*C*_* 55.4, 36.6). This speculation was confirmed by HMBC correlations from H-1 to C-1 (δ*_*C*_* 141.3), C-3 (δ*_*C*_* 147.9), C-4a (δ*_*C*_* 149.0), and C-8a (δ*_*C*_* 116.8); H-4 to C-3 (δ*_*C*_* 147.9), C-5 (δ*_*C*_* 98.4), C-8a (δ*_*C*_* 116.8), and C-9 (δ*_*C*_* 115.8); Me-18 to C-6 (δ*_*C*_* 172.8); H-9 to C-4 δ*_*C*_* 114.5) and C-11 (δ*_*C*_* 131.8); H-10 to C-3 (δ*_*C*_* 147.9) and C-17 (δ*_*C*_* 12.2); H-12 to C-10 (δ*_*C*_* 143.5), C-13 (δ*_*C*_* 35.0), and C-17 (δ*_*C*_* 12.2). ^1^H–^1^H COSY indicated that H-9 had a relationship with H-10, H-12, H-13, and H-14 (me-16); me-15 had a relationship with H-2′and H-3′. Thus, the structure of compound **1** was determined as a new isochromophilone derivative (isochromophilone XIV).

**TABLE 1:** ^1^H NMR and ^13^C NMR data of compounds **1** and **2** in CDCl_3_.

**Position**	**1**		**2**	
	**δ*_*C*_***	**δ*_*H*_*, mult. (*J* in Hz)**	**δ*_*C*_***	**δ*_*H*_*, mult. (*J* in Hz)**
1	141.3, CH	7.86, s	141.1, CH	7.84, s
3	147.9, C		147.4, C	
4	114.5, CH	6.90, s	109.8, CH	7.16, s
4a	149.0, C		144.1, C	
5	98.4, CH	6.82, s	103.2, C	
6	172.8, C		184.7, C	
7	85.4, C		84.7, C	
8	194.1, C		193.9, C	
8a	116.8, C		114.3, C	
9	115.8, CH	5.58,d (15.6)	116.2, CH	5.58,d (15.6)
10	143.5, CH	6.92, d (15.6)	143.1, CH	6.96, d (15.6)
11	131.8, C		131.8, C	
12	148.5, CH	5.65, d (9.7)	147.9, CH	5.66, d (9.7)
13	35.0, CH	2.40, m	35.0, CH	2.40, m
14	30.0, CH_2_	1.31, m; 1.41, m	30.0, CH_2_	1.31, m; 1.40, m
15	11.9, CH_3_	0.83, t (7.4)	12.0, CH_3_	0.85, t (7.4)
16	20.2, CH_3_	0.98, d (6.6)	20.2, CH_3_	0.99, d (6.7)
17	12.2, CH_3_	1.49, s	12.2, CH_3_	1.50, s
18	30.0, CH_3_	1.73, s	23.2, CH_3_	1.60, s
19	170.9, C		170.2, C	
20	106.2, C		20.2, CH_3_	2.19, s
21	194.6			
22	29.1, CH_3_	2.52, s		
1′	140.2, C		140.3, C	
2′	126.4, CH	7.29, m	126.6, CH	7.29, m
3′	130.3, CH	7.56, m	130.1, CH	7.55, m
4′	130.4, CH	7.56, m	130.2, CH	7.55, m
5′	130.3, CH	7.56, m	130.1, CH	7.55, m

Compound **2** (isochromophilone XV) was obtained as a yellow powder, and its molecular formula C_27_H_28_ClNO_4_ was deduced from HRESIMES ion peak at *m*/*z* 466.1816 [M + H]^+^, which was consistent with ^1^H and ^13^C NMR data (Table 1 and Supplementary Figures S10–S15). The ^1^H NMR and ^13^C NMR data were similar to those of isochromophilone IV (Lucas et al., 2007; Wang et al., 2010), except that the imino at position 2 in isochromophilone IV appeared to be aniline, which corresponded to the increase in molecular weight of **2** by 92 amu compared to **4** (δ*_*C*_* 55.4, 36.6). This was further confirmed by the HMBC correlation of H-1 to C-1 (δ*_*C*_* 141.1), C-3 (δ*_*C*_* 147.4), C-4a (δ*_*C*_* 144.1), C-8a (δ*_*C*_* 114.3), and C-8 (δ*_*C*_* 193.9); H-4 to C-3 (δ*_*C*_* 147.4), C-5 (δ*_*C*_* 103.2), C-8a (δ*_*C*_* 114.3), and C-9 (δ*_*C*_* 116.2); Me-18 to C-6 (δ*_*C*_* 184.7) and C-8 (δ*_*C*_* 193.9); H-9 to C-4 (δ*_*C*_* 109.8) and C-1 (δ*_*C*_* 141.1); H-10 to C-3 (δ*_*C*_* 147.4) and C-17 (δ*_*C*_* 12.2); H-12 to C-10 (δ*_*C*_* 143.1), C-13 (δ*_*C*_* 35.0), and C-17 (δ*_*C*_* 12.2); and ^1^H–^1^H COSY correlation of H-9 to H-10, H-12, H-13, H-14, Me-16, and Me-15; H-2′ to H-3′. To our knowledge, this compound was first discovered as a natural product, but reported as semisynthetic derivatives (Wei et al., 2017).

Compound **3** was obtained as a red crystal, and its molecular formula C_21_H_24_ClNO_4_ was deduced from HRESIMES *m*/*z* 390.1518 [M + H]^+^. ^1^H NMR and ^13^C NMR data were as follows: ^1^H NMR (600 MHz, CDCl_3_): δ:7.68 (1H, s, H-1), 6.70 (1H, s, H-4), 6.00 (1H, d, *J* = 16.0 Hz, H-9), 6.82 (1H, d, *J* = 16.0 Hz, H-10), 5.65 (1H, d, *J* = 9.84 Hz, H-12), 2.47 (1H, m, H-13), 1.32 (2H, m, H-14), 0.85 (3H, t, m, H-15), 1.04 (3H, d, *J* = 6.7 Hz, H-16), 1.83 (3H, s, H-17), 1.66 (3H, s, H-18), 2.25 (3H, s, H-20); ^13^C NMR (151 MHz, CDCl_3_) δ: 138.8 (CH, C-1), 147.1 (C, C-3), 110.73 (CH, C-4), 148.25 (C, C-4a), 100.67 (C, C-5), 183.76 (C, C-6), 84.88 (C, C-7), 193.29 (C, C-8), 116.65 (CH, C-9), 143.30 (CH, C-10), 132.04 (C, C-11), 114.67 (CH, C-12), 35.06 (CH, C-13), 30.03 (CH_2_, C-14), 11.95 (CH_3_, C-15), 20.16 (CH_3_, C-16), 12.36 (CH_3_, C-17), 23.7 (CH_3_, C-18), 170.23 (C, C-19), 20.37 (CH_3_, C-20). These showed almost no difference with sclerotioramine (C_21_H_24_ClNO_4_) described in literature (Wang et al., 2010).

Compound **4** was obtained as a yellowish powder, and its molecular formula C_21_H_23_O_5_Cl was deduced from HRESIMES at ion peak at *m*/*z* 389.1325 [M-H]^–^. ^1^H NMR and ^13^C NMR data were as follows: ^1^H NMR (600 MHz, CDCl_3_) δ: 7.92 (1H, s, H-1), 6.63 (1H, s, H-4), 6.08 (1H, d, *J* = 15.72 Hz, H-9), 7.06 (1H, d, *J* = 15.66 Hz, H-10), 5.69 (1H, d, *J* = 9.72 Hz, H-12), 2.47 (1H, m, H-13), 1.42 (2H, m, H-14), 0.85 (3H, t, *J* = 7.26 Hz, H-15), 1.83 (1H, s, H-16), 1.00 (3H, d, *J* = 6.6 Hz, H-17), 1.56 (1H, s, H-18), 2.16 (3H, s, 7-OCOCH_3_); ^13^C NMR (151 MHz, CDCl_3_) δ: 152.67 (CH, C-1), 158.03 (C, C-3), 106.35 (CH, C-4), 138.60 (C, C-4a), 114.51 (C, C-5), 191.74 (C, C-6), 84.53 (C, C-7), 185.90 (C, C-8), 110.78 (C, C-8a), 115.63 (CH, C-9), 142.79 (C, C-10), 131.93 (C, C-11), 148.79 (CH, C-12), 35.10 (CH, C-13), 30.02 (CH_2_, C-14), 11.92 (CH_3_, C-15), 12.33 (CH_3_, C-16), 20.17 (CH_3_, C-17), 22.49 (CH_3_, C-18), 170.04 (COCH_3_), 20.04 (COCH_3_). It indicated that compound **4** was (+) sclerotiorin (C_21_H_23_O_5_Cl) (Chidananda and Sattur, 2007).

### UHPLC-Q-TOF-MS Analysis of Purified Metabolites From Crude Extracts

**FIGURE 3:**
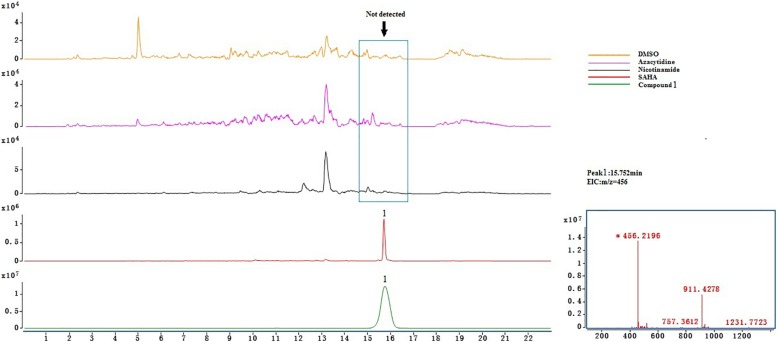
UHPLC-QTOF-MS analysis of compound **1** in different samples.

Crude extract samples were detected by UHPLC-Q-TOF-MS. As a result, compounds **2**, **3**, and **4** existed in all of the samples (Supplementary Figure S16); this suggests that the total of these four ingredients was not the product of SAHA inducing. However, only compound **1** was detected (Figure 3) in the extract of SAHA-treated fermentation broth, but not in the control that showed an intense peak at a retention time of 15.752 min with *m*/*z* of 456.2196[M + H]^+^, which was consistent with purified compound **1** as a standard. This indicated SAHA may activate the dormant gene clusters in the biosynthetic pathways of compound **1**.

### *In vitro* Anti-phytopathogenic Activity

The inhibition activities of compounds **1**–**4** against mycelial growth of nine phytopathogenic fungi were tested *in vitro* (Table 2). The results showed (+) that sclerotiorin (**4**) displayed broad and important inhibition activities against *C. lunata* (IC_50_ = 2.1 μg/mL), *C. clavata* (IC_50_ = 21.0 μg/mL), *F. oxysporum* f. sp. *mornordicae* (IC_50_ = 40.4 μg/mL), and *B. dothidea* (IC_50_ = 27.8 μg/mL), which were comparable to those of referenced cycloheximide with IC_50_ values of 0.3, 12.4, and 15.3 μg/mL, respectively. Ingredient **3** exhibited a selective inhibition activity against *C. graminicola* with an IC_50_ value of 9.7 μg/mL, which was comparable to that of cycloheximide (IC_50_ = 1.8 μg/mL). Meanwhile, metabolite **2** showed a relatively weak activity against *C. graminicola* with an IC_50_ value of 29.9 μg/mL.

**TABLE 2:** The IC_50_ values of compounds **1**–**4** against the tested phytopathogens (in μg/mL).

**Phytopathogens**	**1**	**2**	**3**	**4**	**Cycloheximide^a^**
*C. graminicola*	> 50 ± 0	29.9 ± 0.1	9.7 ± 0.1	> 50 ± 0	1.8 ± 0.1
*C. lunata*	> 50 ± 0	> 50 ± 0	> 100 ± 0	2.1 ± 0.1	0.3 ± 0.1
*G. zeae*	> 50 ± 0	> 50 ± 0	> 50 ± 0	> 50 ± 0	3.3 ± 0.2
*C. clavata*	> 50 ± 0	> 50 ± 0	> 100 ± 0	21.0 ± 0.1	5.0 ± 0.1
*A. solani*	> 50 ± 0	> 50 ± 0	> 100 ± 0	> 50 ± 0	0.9 ± 0.1
*C. cassiicola*	> 50 ± 0	> 50 ± 0	> 50 ± 0	> 50 ± 0	2.6 ± 0.1
*F. oxysporum* f. sp. *cucumerinum*	> 50 ± 0	> 50 ± 0	> 50 ± 0	> 50 ± 0	4.9 ± 0.2
*F. oxysporum* f. sp. *mornordicae*	> 50 ± 0	> 50 ± 0	> 100 ± 0	40.4 ± 0.2	12.4 ± 0.2
*B. dothidea*	> 50 ± 0	> 50 ± 0	> 100 ± 0	27.8 ± 0.1	15.3 ± 0.2

*Results were presented as the mean standard deviation for triplicate experiments. ^*a*^Cycloheximide was used as the positive control.*

### *In vitro* Anti-bacterial and Anti-yeast Activities

Compounds **1**–**4** were tested for their antibacterial and anti-yeast activity against a panel of representative pathogens. As shown in Table 3, both compounds **3** and **4** exhibited potent activities against *B. subtilis*, with the same ZOI values of 9.10 mm compared with that of positive gentamycin (ZOI = 10.80 mm). Notably, metabolite **1** displayed a moderate activity against *C. albicans* with a ZOI value of 10.50 mm, compared with that of referenced amphotericin B (ZOI = 23.20 mm).

**TABLE 3:** ZOI (mm) of compounds **1**–**4** against the tested strains.

**Compounds**	***E. coli***	***S. aureus***	***B. subtilis***	***C. albicans***
1	2.95 ± 0.01	3.00 ± 0.27	NI	10.50 ± 0.07
2	NI^b^	NI	NI	NI
3	NI	4.10 ± 0.01	9.10 ± 0.30	NI
4	NI	5.80 ± 0.4	9.10 ± 1.70	NI
PC^a^	27.70 ± 1.10	32.30 ± 1.20	10.80 ± 1.70	23.20 ± 0.70

*^*a*^PC: gentamicin was used as a positive control for bacteria; amphotericin B was used as a positive control for yeast. Results were presented as the mean standard deviation for triplicate experiments. ^*b*^NI, not inhibited.*

## Discussion

Sclerotiorin, first isolated from *Penicillium sclerotiorum*, belongs to the azaphilone-type family of natural products, with a γ-lactone, a conjugated ketone, a chlorine atom at C-5, and a branched C-7 side chain (Maccurin and Reilly, 1940). Sclerotiorin and its derivatives were mostly isolated from *Penicillium* species from soil (Arai et al., 1995; Nam et al., 2000; Celestino et al., 2014), sea (Cheung et al., 2014; Chen et al., 2017), and plant endophytes (Giridharan et al., 2012). However, to the best of our knowledge, it was the first reported that the two new natural products **1** and **2** and the following metabolites **3** and **4** were obtained from the title strain *P. mallochii* CCH01, a fungus residing in the gut of *E. oblique.*

Azacytidine, a DNA methylation-modifying agent, was verified as an effective epigenetic modifier that altered secondary metabolites of an Atlantic-forest-soil-derived *P. citreonigrum* and led to the production of six azaphilones (Wang et al., 2010). As far as we know, there are no references about new azaphilones obtained from *Penicillium* treated with HDAC inhibitors. In the present study, the effect of three different epigenetic modifiers, i.e., 5-azacytidine, SAHA, and nicotinamide on the metabolic profile of *P. mallochii* CCH01 was studied. Interestingly, a significant variation in the metabolome of the fungus was observed when treated with SAHA, an HDAC inhibitor. On the basis of further LC-MS analysis experiments, metabolites **2–4** were detected in the crude extracts of both control and treated cultures; only compound **1** was found exclusively in the extract of SAHA-treated fungus (Figure 3). This indicated that the epigenetic modifier SAHA can be used to generate new azaphilones from the gut fungus *P. mallochii* CCH01.

## Conclusion

Here, one strain identified as *P. mallochii* CCH01 was isolated from the gut of *E. oblique*, a pest living on tea plants. For developing the potential of *P. mallochii* CCH01 for the production of new compounds, three chemical epigenetic reagents were applied to stimulate the strain. As a result, SAHA brought significant changes in the secondary metabolites of the fungus. Subsequently, four compounds, including 2 new isochromophilone derivatives, were isolated from the culture treated with SAHA. This result suggests that SAHA can be used to identify diverse natural products hidden in silent biosynthetic pathways from gut fungus *P. mallochii* CCH01. Sclerotiorin (**4**) possessed a good prospect in crop and plant protection, with important activities against phytopathogenic fungi. Interestingly, as two simple amine derivatives of sclerotiorin (**4**), both metabolites **2** and **3** exhibited modest inhibition activities against *C. graminicola*, which were different with sclerotiorin. Additionally, the new compound **1** showed a promising activity against *C. albicans*, which was comparable to that of referenced amphotericin B. Ingredients **3** and **4** possessed strong antibacterial activities against *B. subtilis in vitro*. Thus, our results highlight the potential of epigenetic modification as powerful strategies for generating the production of cryptic fungal metabolites.

## Data Availability Statement

The raw data supporting the conclusions of this manuscript will be made available by the authors, without undue reservation, to any qualified researcher.

## Author Contributions

YZ and CW designed the research. YZ supervised the study. SZ, HF, and JH performed the experiments and analyzed the data. SZ wrote the manuscript together with CY. All authors revised the manuscript and approved the final version for submission.

## Conflict of Interest

The authors declare that the research was conducted in the absence of any commercial or financial relationships that could be construed as a potential conflict of interest.
