# Supplementary material for: Antimicrobial Metabolites Produced by Penicillium mallochii CCH01 Isolated From the Gut of Ectropis oblique, Cultivated in the Presence of a Histone Deacetylase Inhibitor
Source: Front Microbiol. 2019 Oct 2;10:2186. doi: 10.3389/fmicb.2019.02186 (PMC6783908; doi:10.3389/fmicb.2019.02186)
Supplement: Supplementary file 1 [file Data_Sheet_1.pdf]

Supplementary Materials:

Antimicrobial Metabolites Isolated from *P. mallochii* CCH01, a Gut fungus of *Ectropis oblique* Induced by a Histone Deacetylase Inhibitor

Shuxiang Zhang[1†], Han Fang[1†], Caiping Yin[1], Chaoling Wei[2*], Yinglao Zhang[1*]

1 School of Life Science, Anhui Agricultural University, 130 West Changjiang Rd., Hefei 230036 Anhui, China

2 State Key Laboratory of Tea Plant Biology and Utilization, Anhui Agricultural University, 130 West Changjiang Rd., Hefei 230036 Anhui, China

Correspondence to: Prof. Yinglao Zhang

School of Life Science, Anhui Agricultural University, 130 West Changjiang Rd., Hefei 230036 Anhui, China

Email: zhangyl@ahau.edu.cn;

Tel.: +86-551-6578-6129

Supporting information description

Figure S1. Overview of colony and conidiophores characters of CCH01 in PDA medium.

Figure S2. TLC analysis of epigenetic modifying compounds on the metabolite production of *P. mallochii* CCH01

Figure S3. ^{1}H NMR spectrum of compound **1** at 600 MHz in $CDCl_3$.

Figure S4. ^{13}C NMR spectrum of compound **1** at 150 MHz in $CDCl_3$.

Figure S5. ^{1}H-^{1}H COSY spectrum of compound **1** at 600 MHz in $CDCl_3$.

Figure S6. HMQC spectrum of compound **1** at 600 MHz in $CDCl_3$.

Figure S7. DEPT spectrum of compound **1** at 150 MHz in $CDCl_3$.

Figure S8. HMBC spectrum of compound **1** at 600 MHz in $CDCl_3$

Figure S9. ESI mass spectra of compound **1.**

Figure S10. ^{1}H NMR spectrum of compound **2** at 600 MHz in $CDCl_3$.

Figure S11. ^{13}C NMR spectrum of compound **2** at 150 MHz in $CDCl_3$.

Figure S12. ^{1}H-^{1}H COSY spectrum of compound **2** at 600 MHz in $CDCl_3$.

Figure S13. HMQC spectrum of compound **2** at 600 MHz in $CDCl_3$.

Figure S14. HMBC spectrum of compound **2** at 600 MHz in $CDCl_3$.

Figure S15. ESI mass spectra of compound **2**.

FigureS16. EIC analysis of compounds **2-4** in different samples.

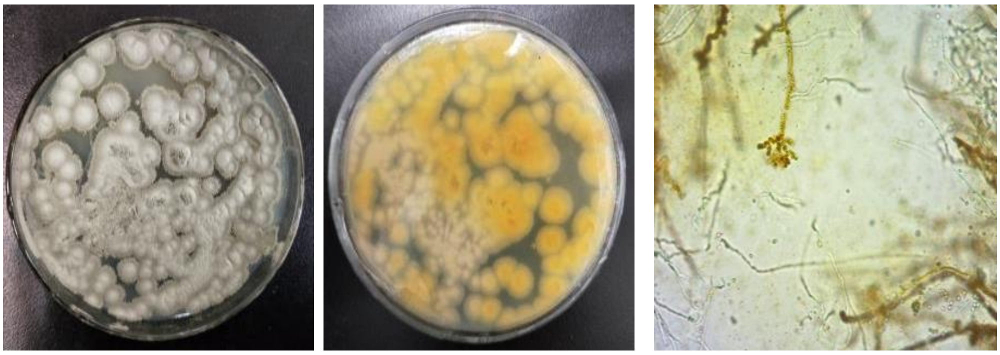

Figure S1 Overview of colony and conidiophores characters of CCH01 in PDA medium.

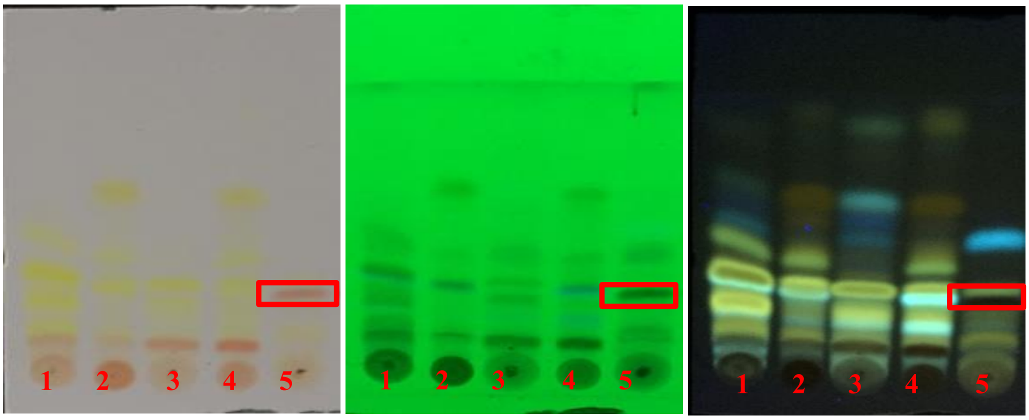

Figure S2 TLC analysis of epigenetic modifying compounds on the metabolite production of *P. mallochii* CCH01

Note: A：nature light　B: 254 nm　C:365 nm

1: CK; 2: DMSO; 3:nicotinamide ;4:5-azacytidine ;5: SAHA

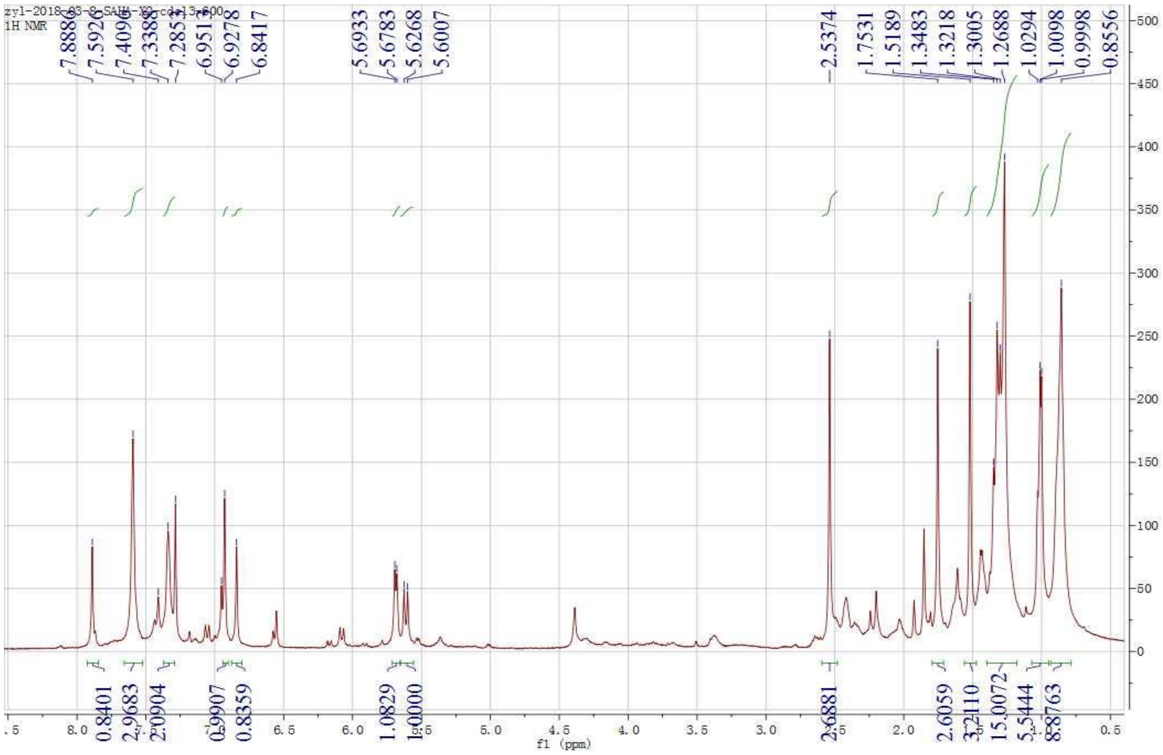

Figure S3. ^{1}H NMR spectrum of compound **1** at 600 MHz in $CDCl_3$.

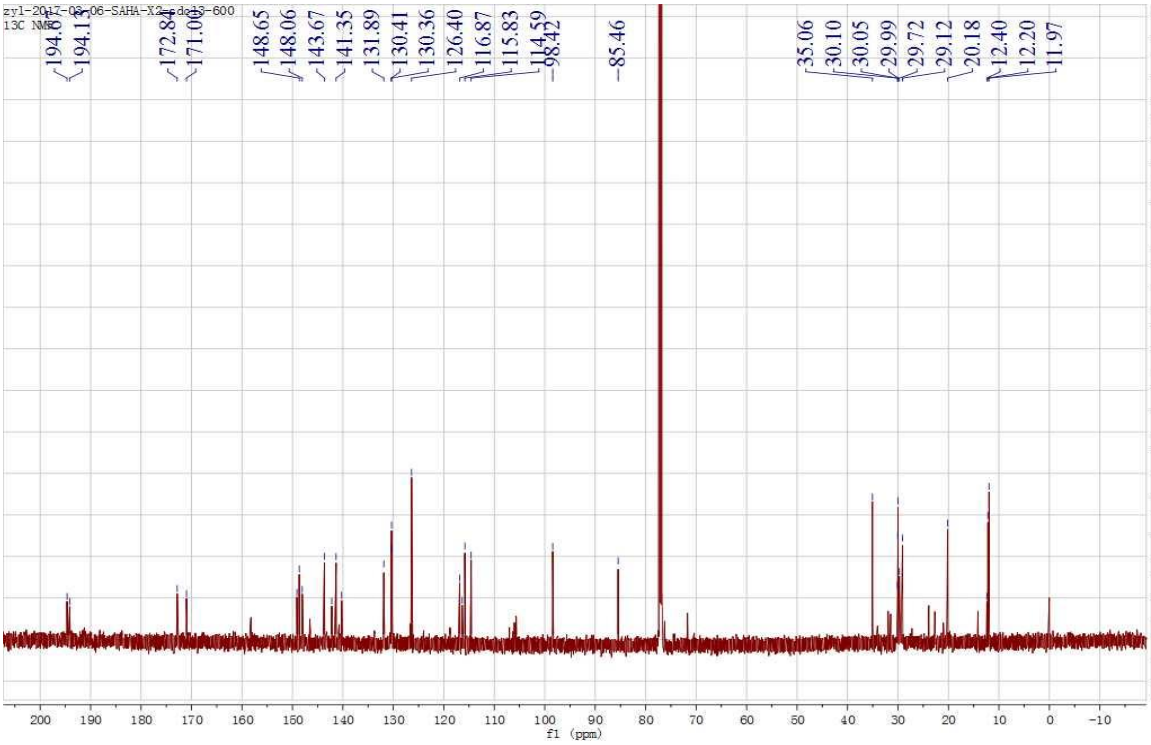

Figure S4. ^{13}C NMR spectrum of compound **1** at 150 MHz in $CDCl_3$.

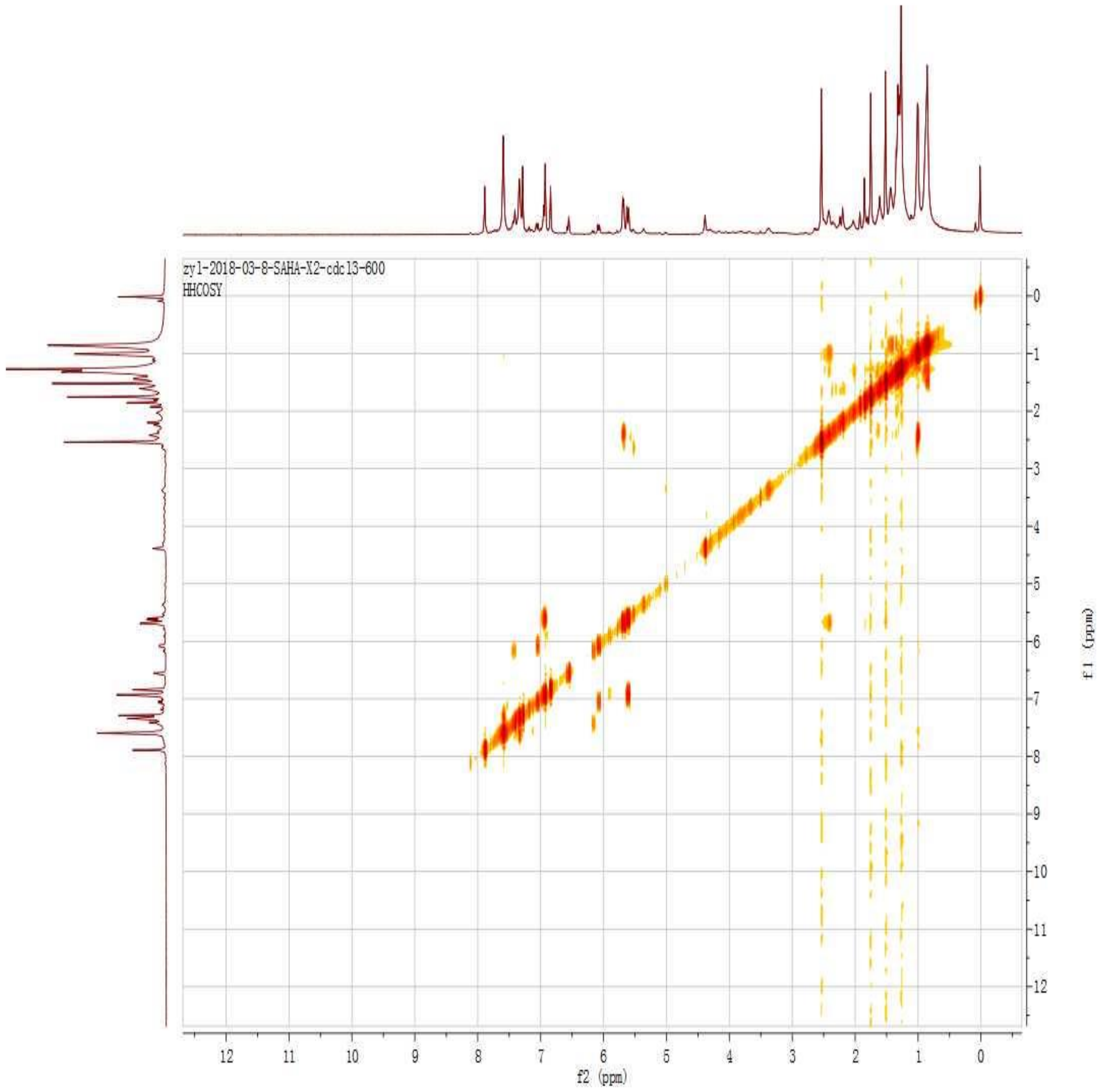

Figure S5. ^{1}H-^{1}H COSY spectrum of compound **1** at 600MHz in $CDCl_3$.

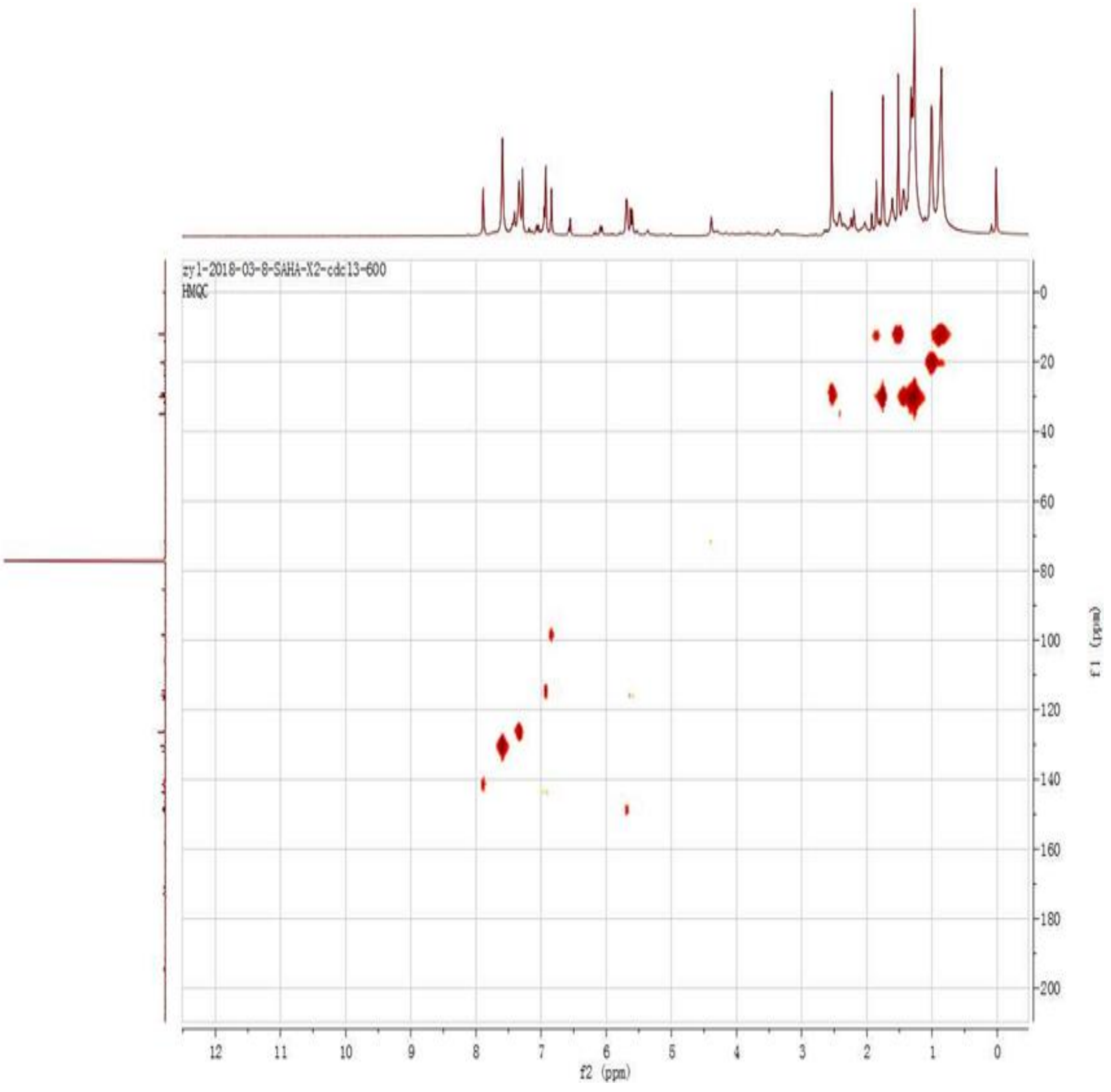

Figure S6. HMQC spectrum of compound **1** at 600 MHz in $CDCl_3$.

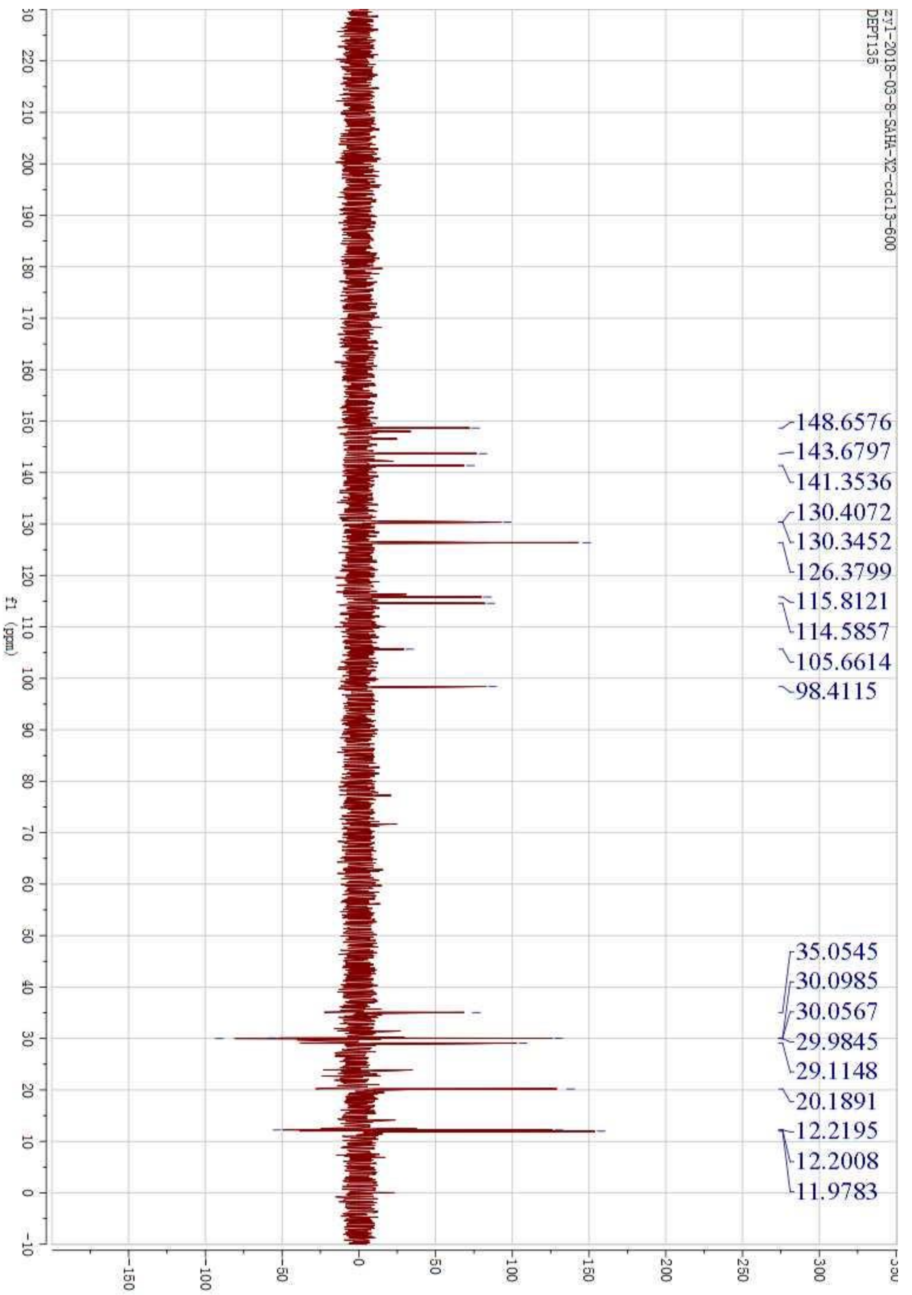

Figure S7. DEPT spectrum of compound **1** at 150 MHz in $CDCl_3$.

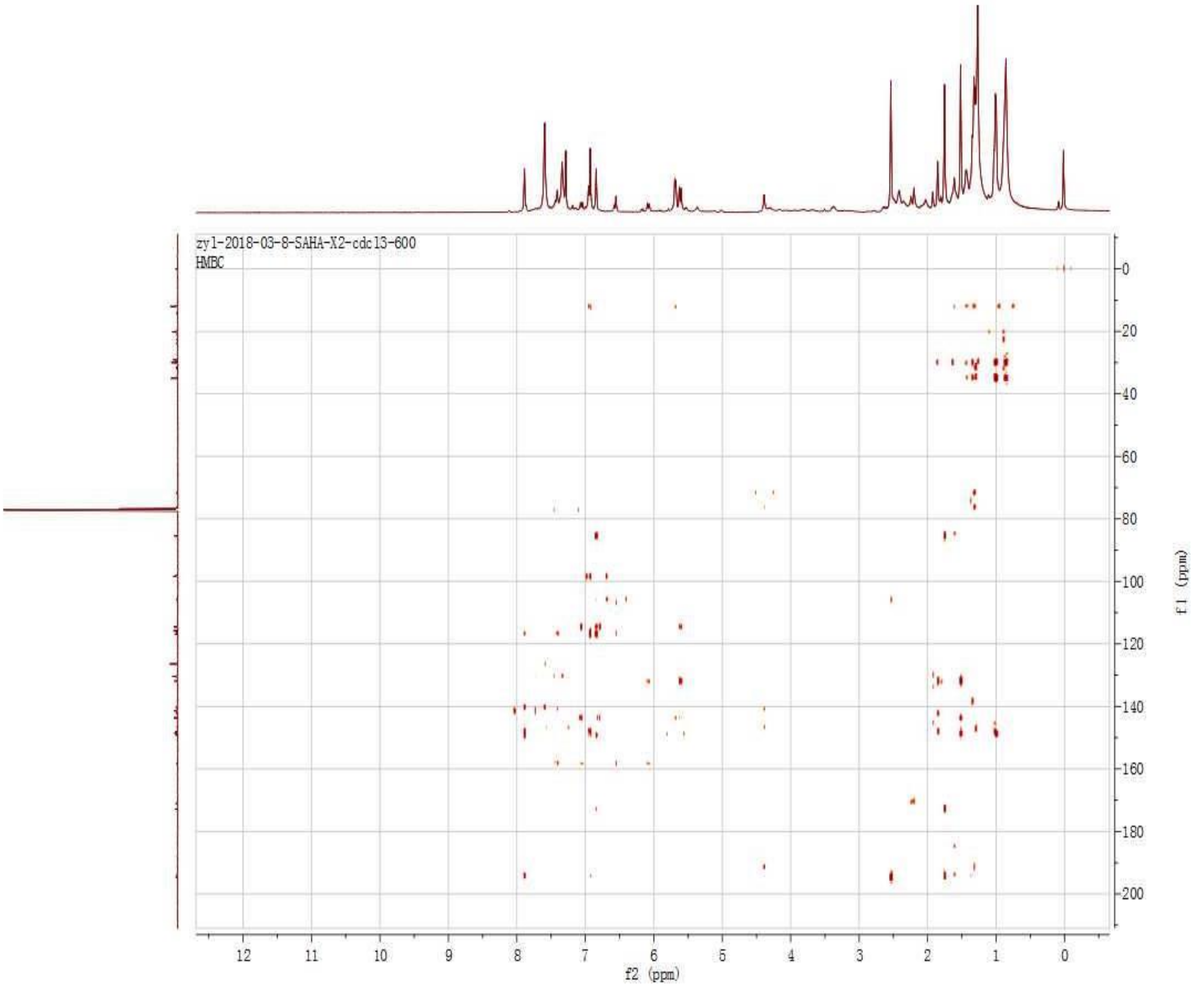

FigureS8. HMBC spectrum of compound **1** at 600 MHz in CDCl3.

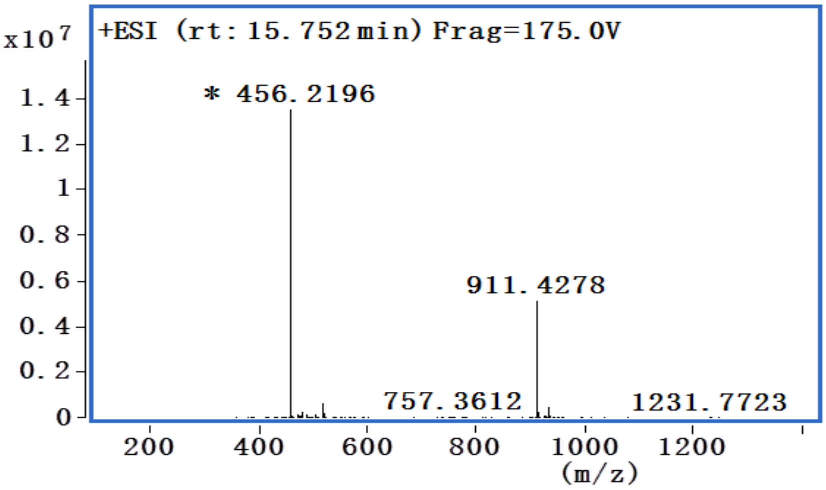

FigureS9. ESI mass spectra of compound **1**.

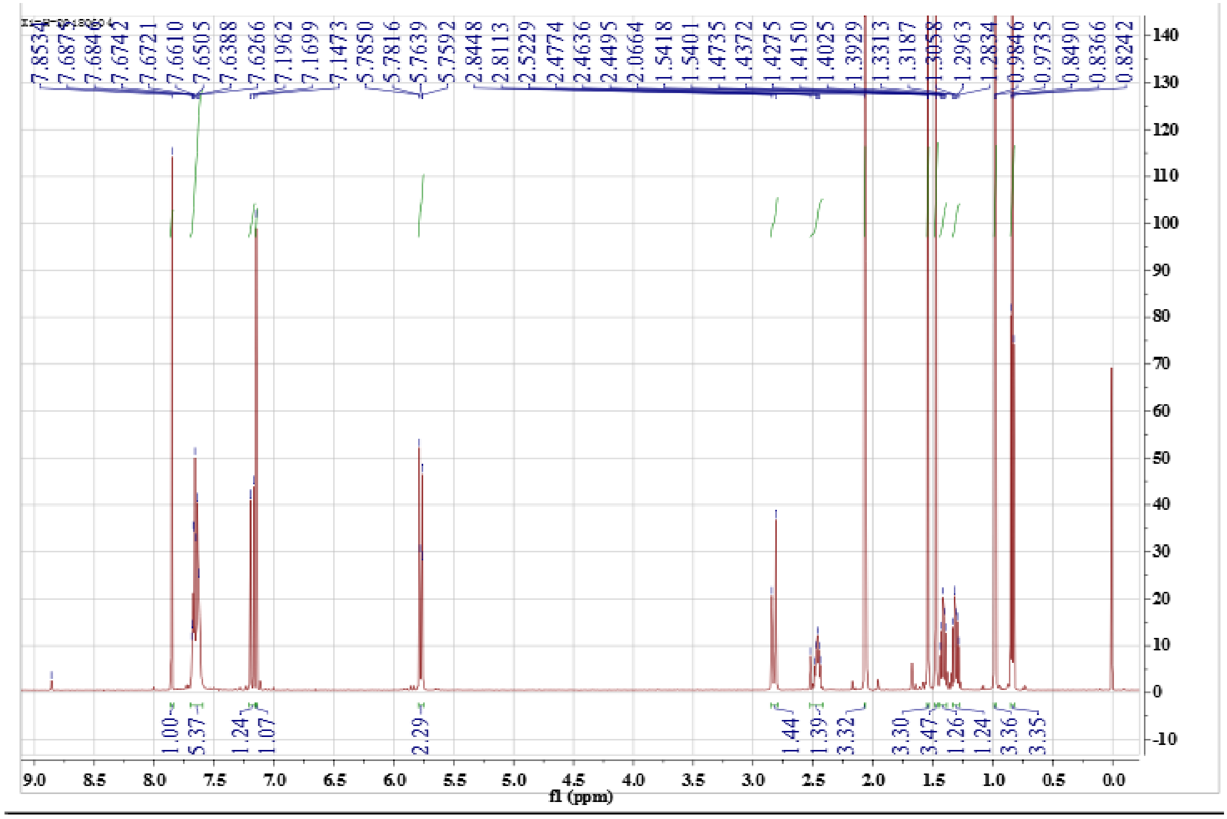

Figure S10. ^{1}H NMR spectrum of compound **2** at 600 MHz in $CDCl_3$.

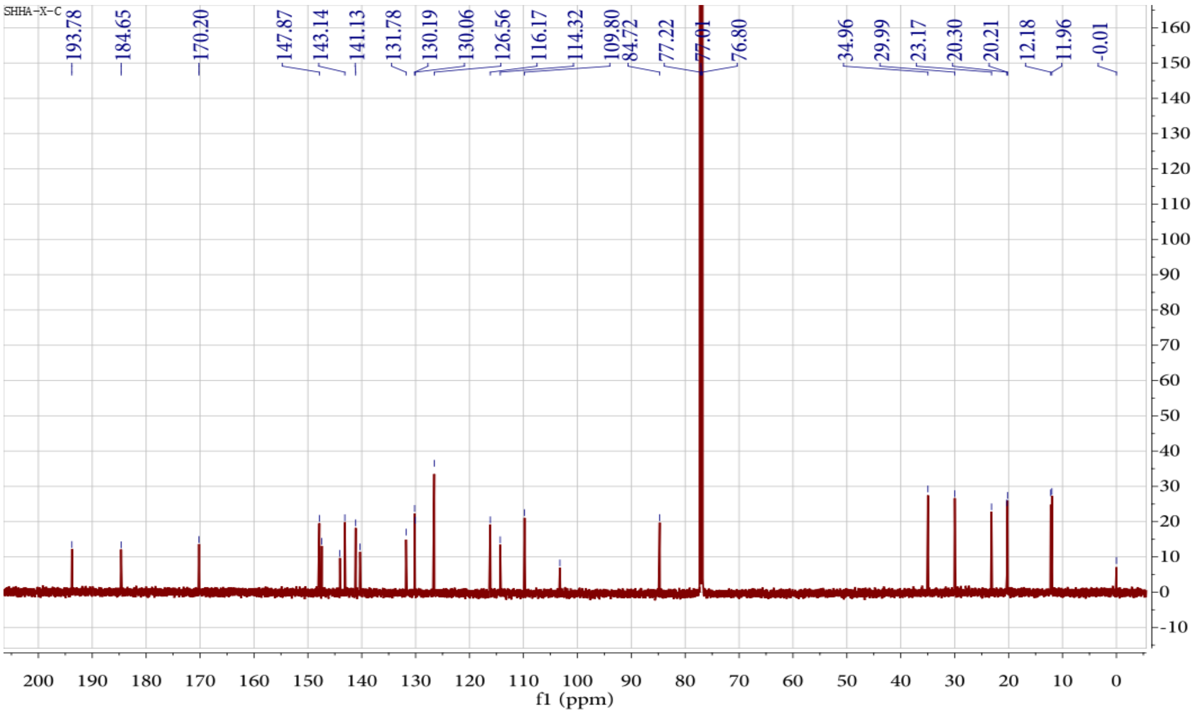

Figure S11. ^{13}C NMR spectrum of compound **2** at 150 MHz in $CDCl_3$.

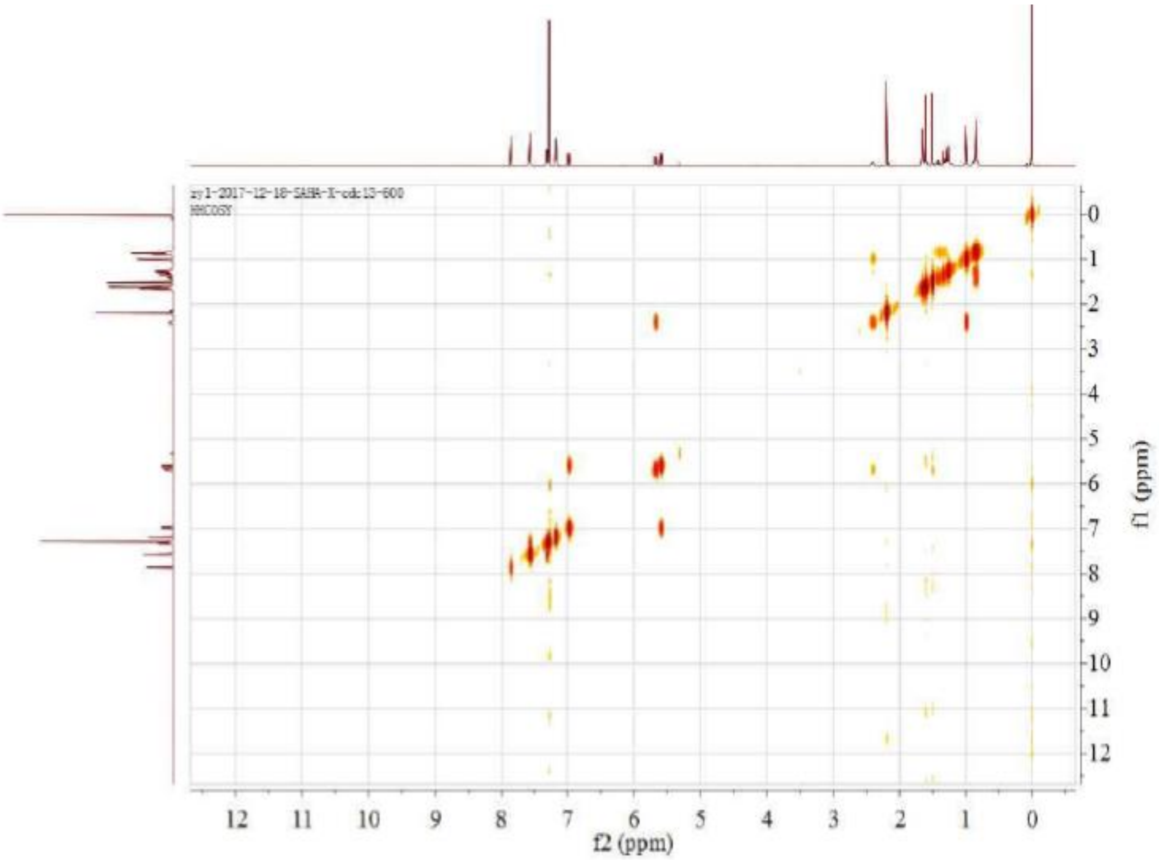

Figure S12. ^{1}H-^{1}H COSY spectrum of compound **2** at 600 MHz in $CDCl_3$.

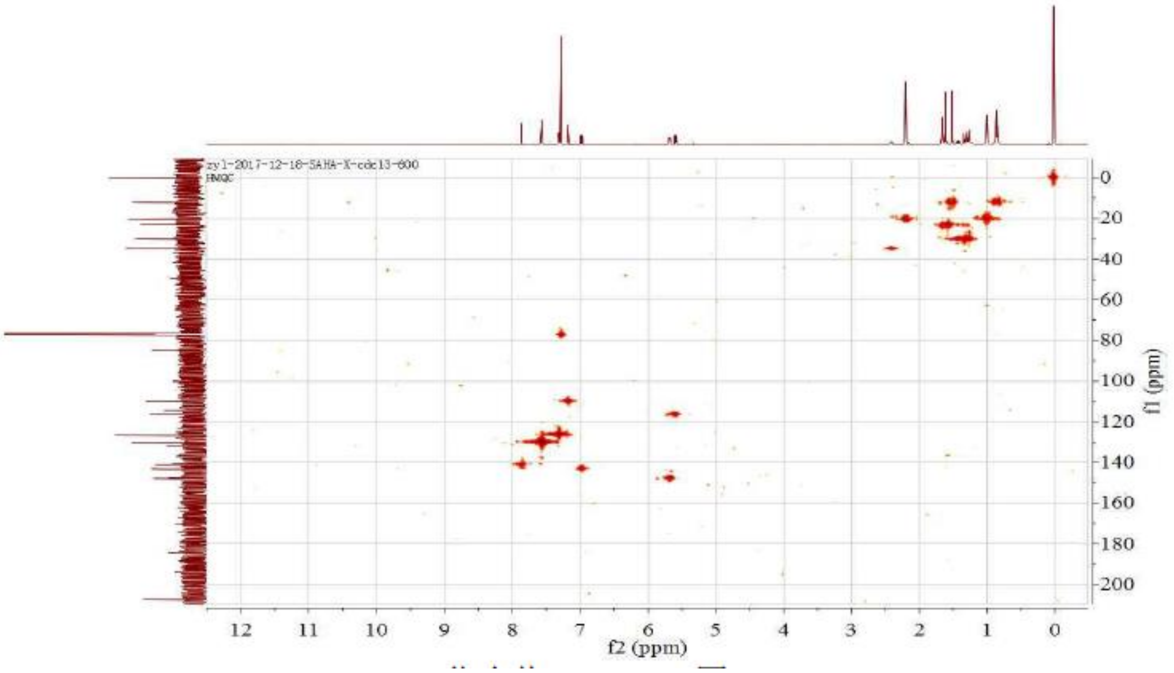

Figure S13. HMQC spectrum of compound **2** at 600 MHz in $CDCl_3$.

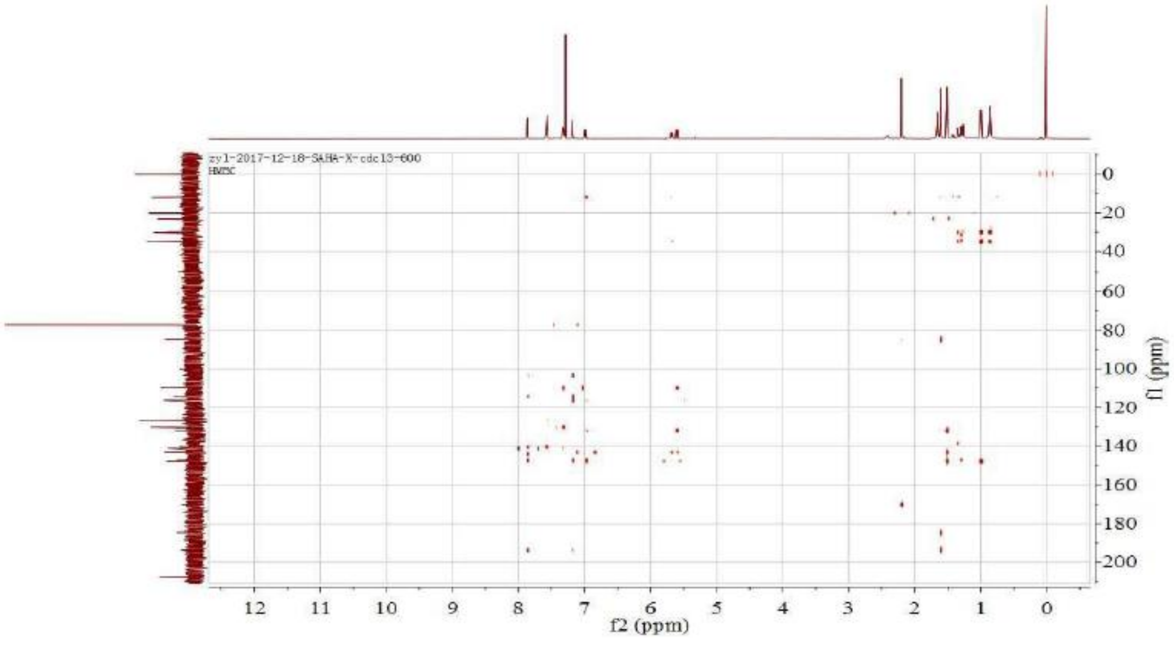

Figure S14. HMBC spectrum of compound **2** at 600 MHz in $CDCl_3$.

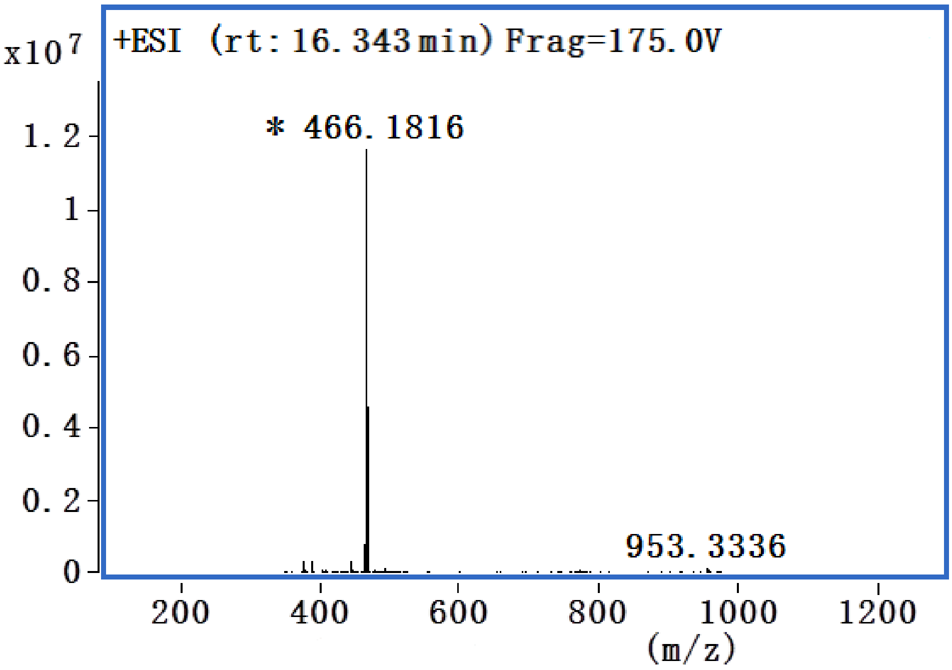

FigureS15**.** ESI mass spectra of compound **2**.

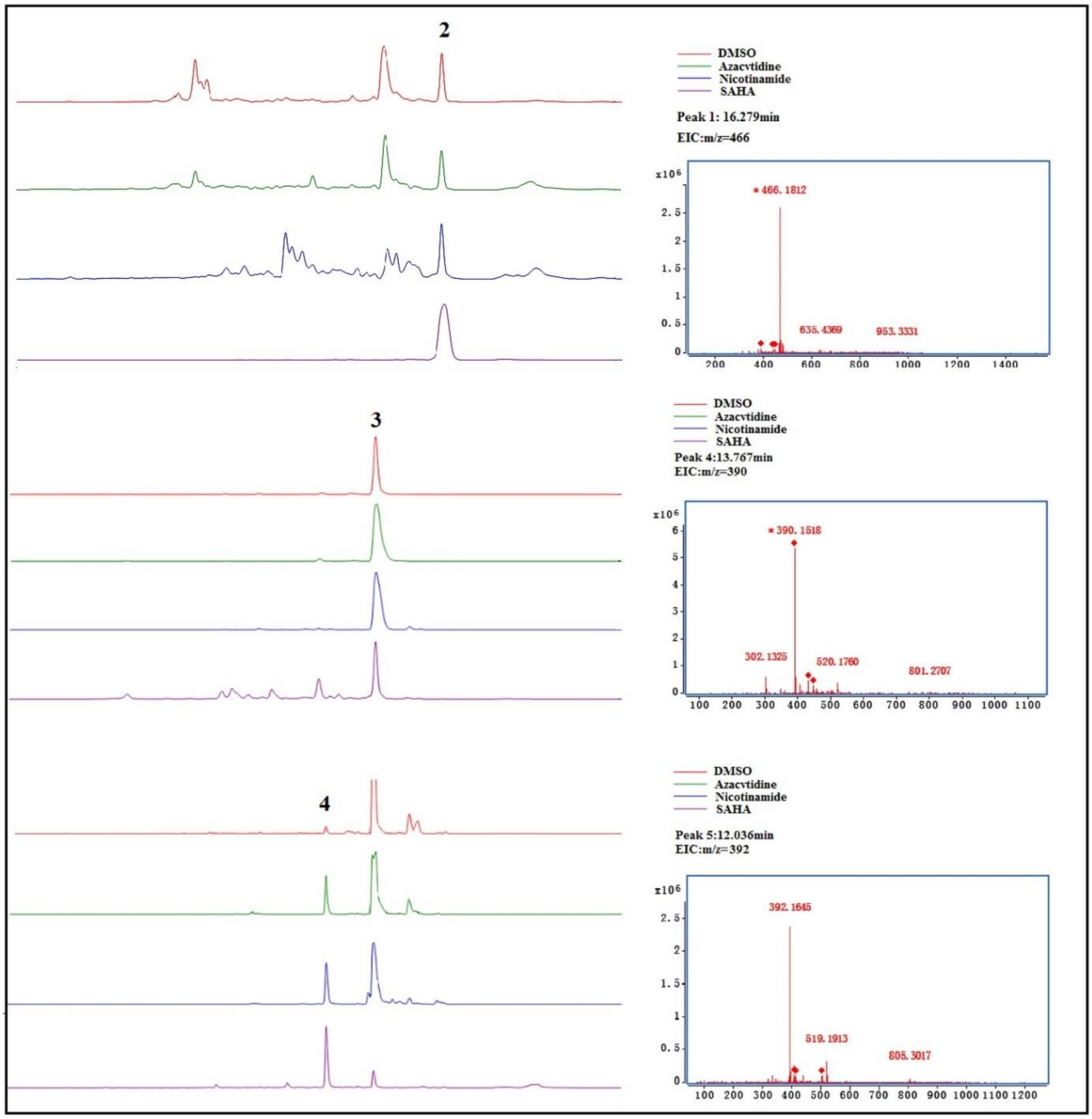

FigureS16. EIC analysis of compounds **2-4** in different samples.
